# Preconceptional paternal caloric restriction of high-fat diet-induced obesity in Wistar rats dysregulates the metabolism of their offspring via AMPK/SIRT1 pathway

**DOI:** 10.1186/s12944-024-02161-6

**Published:** 2024-06-08

**Authors:** Rachakatla Anuradha, M. Srinivas, M. Satyavani, K. Suresh, MN. Muralidhar, Kalashikam Rajender Rao

**Affiliations:** 1 Animal Facility, ICMR-National Institute of Nutrition, Tarnaka, Hyderabad, 500007 Telangana India; 2 ICMR-Centre for Research, Management and Control of Haemoglobinopathies (ICMR- CRMCH), Chandrapur, 442406 Maharashtra India

**Keywords:** Paternal caloric restriction, Metabolic disorders, Male fertility, SIRT1 and AMPK.

## Abstract

**Background:**

Obesity is a metabolic syndrome where allelic and environmental variations together determine the susceptibility of an individual to the disease. Caloric restriction (CR) is a nutritional dietary strategy recognized to be beneficial as a weight loss regime in obese individuals. Preconceptional parental CR is proven to have detrimental effects on the health and development of their offspring. As yet studies on maternal CR effect on their offspring are well established but paternal CR studies are not progressing. In current study, the impact of different paternal CR regimes in diet-induced obese male Wistar rats (WNIN), on their offspring concerning metabolic syndrome are addressed.

**Methods:**

High-fat diet-induced obese male Wistar rats were subjected to caloric restriction of 50% (HFCR-I) and 40% (HFCR-II) and then they were mated with normal females. The male parent’s reproductive function was assessed by sperm parameters and their DNMT’s mRNA expression levels were also examined. The offspring’s metabolic function was assessed by physiological, biochemical and molecular parameters.

**Results:**

The HFCR-I male parents have shown reduced body weights, compromised male fertility and reduced DNA methylation activity. Further, the HFCR-I offspring showed attenuation of the AMPK/SIRT1 pathway, which is associated with the progression of proinflammatory status and oxidative stress. In line, the HFCR-I offspring also developed altered glucose and lipid homeostasis by exhibiting impaired glucose tolerance & insulin sensitivity, dyslipidemia and steatosis. However, these effects were largely mitigated in HFCR-II offspring. Regarding the obesogenic effects, female offspring exhibited greater susceptibility than male offspring, suggesting that females are more prone to the influences of the paternal diet.

**Conclusion:**

The findings highlight that HFCR-I resulted in paternal undernutrition, impacting the health of offspring, whereas HFCR-II largely restored the effects of a high-fat diet on their offspring. As a result, moderate caloric restriction has emerged as an effective weight loss strategy with minimal implications on future generations. This underscores the shared responsibility of fathers in contributing to sperm-specific epigenetic imprints that influence the health of adult offspring.

**Supplementary Information:**

The online version contains supplementary material available at 10.1186/s12944-024-02161-6.

## Introduction

Obesity arises from an abnormal accumulation of body fat, which can lead to various adverse health effects, making it the fifth most significant contributor to global mortality. It is widely recognized as a global epidemic, often referred to as “globesity,” and is associated with significant health risks such as CVD, type 2 diabetes mellitus, kidney disease etc., contributing to a decline in the overall quality of life worldwide [1, 2].

Caloric restriction (CR) is a nutritional weight loss strategy with less calorie intake while maintaining proper nutritional requirements. Investigations on a variety of species have shown that it can increase longevity, slow the rate of biological aging and prevent chronic metabolic disorders [3]. The CR is thought to modulate inflammation, and lipid & glucose metabolism through sirtuin signaling, contributing to the extension of the mammalian lifespan. Among the seven mammalian sirtuins, SIRT1 plays a crucial role in cellular metabolism and determining cell fate during CR [4, 5]. SIRT1, an NAD+ -dependent deacetylase targets many transcription factors like PGC1α, PPARα, PPARγ and SERBP1c etc., in numerous tissues including the liver, adipose and muscle [6, 7].

Further, CR results in the upregulation of AMPK signaling, and its downstream regulators such as SIRT1 and PGC1α [8, 9]. AMPK is a heterotrimeric protein kinase in mammals that is encoded by two α, two β, and three γ subunits, together these subunits produce 12 distinct αβγ isoforms [10]. SIRT1 activity also appears to be under the control of adiponectin whose levels are also improved directly by CR [11].

Studies have suggested that parental CR before or around the time of conception can lead to epigenetic changes that significantly affect fetal growth, development, and the long-term health of offspring. While much attention has been focused on the nutritional and environmental factors affecting mothers, emerging data from both humans and rodents demonstrate that the nutritional status of fathers before conception can also influence the health and metabolism of their offspring [12–15]. The transmission of paternal nutritional sensitive programming to the offspring is simpler either by sperm and/or seminal plasma than that from mothers to children and the programming of lipid metabolism is passed on to future generations through sperm [16]. Paternal long-term exposure to physiological stress carries the epigenetic memory of sperm that affects future generations through embryonic reprogramming [17]. Paternal diet-induced obesity alters epigenetics by programming SETD2 gene methylation in the F_0_ and F_1_ generations [18]. Preconceptional paternal high-fat diet exposure leads to dysregulation of lipid & triglyceride metabolism and fatty liver conditions in their offspring, indicating that obesity is transferred from fathers to their offspring [19]. Studies on rodents have found that TM6SF2 is a novel genetic player associated in the development of nonalcoholic fatty liver disease by regulating lipid metabolism thus involved in cardiometabolic disease [20, 21]. A paternal high-fat diet alters glucose metabolism by modifying gluconeogenesis through changes in igf2/h19 DNA methylation in offspring [22]. Paternal obesity induced by high fat also programs inflammation and oxidative stress in their sperm and testes, which alter the reproductive function of male offspring due to disturbances in the hypothalamic-pituitary-gonadal axis [23]. Fathers’ low-protein diet has shown intergenerational effects on offspring metabolism through the modification of ATF7, an epigenetic regulator [24]. Paternal malnutrition leads to reduced reproductive function via increased oxidative stress in the testes and sperm, which leads to increased obesogenic potencies in offspring at an early age [13, 25–27].

Despite the existing research, there is a lack of exploration into the effects of CR on the health of offspring in diet-induced obese males. Further, it is essential to elucidate the mechanisms involved in paternal CR in obese individuals to better understand the pathophysiology of human obesity. In the present study, the effects of different CR regimes on the reproductive health of high-fat diet-induced obese male Wistar rats and the different outcomes in their offspring related to inflammation and lipid and glucose metabolism via the AMPK/SIRT1 pathway are reported. This information is needed at this lifestyle change juncture to design nutritional strategies for fathers that can help us save our children from metabolic syndrome burden.

## Methodology

### Animal experimental design

The study involved male Wistar (WNIN) rats (*n* = 24). After weaning, these rats were parted into control (*n* = 6) and high-fat (*n* = 18) groups. These groups were given control or high-fat diets (60%) ad libitum for 12 weeks respectively. Subsequently, the high-fat group was subdivided into three groups (*n* = 6 each): the HF group continued on a high-fat diet, the HFCR-I group (transferred to a control diet with 50% caloric restriction) and the HFCR-II group (transferred to a control diet with 40% caloric restriction) for the next 8 weeks. The control group was fed control diet since its inception. The caloric restrictions of 50% and 40% were determined based on the previous week’s food consumption during the ad libitum period. After caloric restriction, all male rats were subjected to mating with healthy age-matched female rats fed ad libitum on a control diet to produce the F_1_ generation. After weaning, the offspring of all the male parents were fed a control diet for 8 weeks (Supplementary Fig. 1).

The rats were individually housed and given 24-hour access to water and kept in sterile polycarbonate grill cages throughout the experiment. The standard conditions included a temperature of 22 ± 2 °C, humidity of 55 ± 5%, an air exchange rate of 14–16 per hour, 12 h of light and 12 h of darkness to maintain day and night cycles [28]. After successful mating, F_0_ and F_1_ generation animals were euthanized following an overnight fast by carbon dioxide euthanasia. The metabolically active tissues such as blood, liver, adipose and other tissues were collected, frozen in liquid nitrogen quickly and stored at -80 °C. Portions of the liver and testes were stored in 10% formalin for histopathology investigations.

### Animal diet

The composition of the control diet was the same as that of the AIN 93G diet according to Reeves et al. 1997. The energy levels of the control diet were 395 kcal/100 g, where the energy contributed from carbohydrates was 64%, the protein content was 20%, and the fat content was 16%. The experimental high-fat diet consisted of a modified AIN 93G diet in which the energy contributed by the fat was adjusted to 60% by using lard as a fat source to induce obesity in the experimental animals. The energy levels of a high-fat diet were 540 kcal/100 g of diet, where the energy contributed from carbohydrates was 20%, the protein was 20% and the fat was 60% [28–30]. (Supplementary Table 1).

### Physiological methods

Food intake was measured every day from the beginning of the experiment, the body weights were measured on a weekly basis, and the birth weights of the offspring were measured on an electronic weighing balance. Anthropometric measurements such as energy intake, FER and BMI were calculated by using the formulas described in our previous study [2]. The body composition of the rats was assessed by DEXA (SCAN Type: A Rat WB, Model: Discovery A. S/N 82,382, Multidetector, Springfield, USA). The DEXA reports were used to calculate total body fat %, LBM %, and FFM % by using the formulas explained in a previous study [2]. The organ and WAT weights (retroperitoneal fat, omental fat, mesenteric fat and gonadal fat) were taken after the animals were euthanized, and the AI was calculated by using the formula AI= (WAT weight x100)/ (body weight-WAT weight) [31–33].

### Biochemical methods

Plasma adiponectin (Millipore, USA. Cat # RAB1136-1KT) and insulin (Millipore, USA. Cat # RAB0904-1KT) were assessed by sandwich ELISA following the manufacturer’s protocol. Enzyme-based commercially available assay kits (Biosystems S.A., Spain) were used to measure total cholesterol (Cat # 11,505), triglycerides (Cat # 11,528) and HDL-cholesterol (Cat # 11,648) in circulation following the instructions provided by the manufacturer. Stress parameters such as TAC and catalase activity were determined in plasma using a spectrophotometric approach following the instructions given in the kits, which were purchased from BioAssay Systems, USA (Cat # DTAC-100 and # ECAT-100). Blood testosterone levels were analyzed by ELISA following the instructions provided with the kit (DRG, Cat # EIA-5179). Rat cytokine/chemokine profiles, including those of the rat adipocytokine leptin, interleukin-6 (IL-6), interleukin-1 beta (IL-1β), monocyte chemoattractant protein-1 (MCP-1, CCL2) and tumor necrosis factor-alpha (TNF-α), were analyzed in plasma samples with a Milliplex rat-cytokine immunoassay kit (Millipore, USA). Cat # RECYTMAG-65 K-08) according to the manufacturer’s instructions.

### Insulin resistance

Insulin resistance was assessed by using the HOMA-β and HOMA-IR formulas from fasting insulin and glucose levels where HOMA-IR= [fasting insulin (µIU/mL) × fasting glucose (mmol/L)]/22.5, while HOMA-β= [20 × fasting insulin (µIU/mL)]/[fasting glucose (mmol/L) − 3.5] [34, 35].

### OGTT

Rats were orally challenged with 40% w/v dextrose solution (2.5 g/kg of body weight) to determine the rate of blood glucose clearance in circulation following an overnight fast. The glucose levels in the blood obtained from the supraorbital region at intervals of 0, 30, 60, and 120 min while under mild isoflurane were measured by a glucometer [35, 36]. The trapezoidal approach was used to calculate the AUC for glucose [37].

### Histopathology

Liver and testicle sections preserved in 10% buffered formalin were fixed in paraffin blocks, and 4 μm tissue slices were cut using a microtome. The slices were deparaffinized, rehydrated, and then stained with hematoxylin for 20–40 min. The slides were cleaned of the extra dye by utilizing 70% ethanol and 1% hydrochloric acid. The slides were then stained with eosin for 10 min, followed by cleaning in 100% ethanol for five minutes. The slides were finally cleaned with xylene, mounted with a cover slip, and allowed to dry for an entire night. An inverted microscope (Cilika/BT/Invidigital Inverted Trinocular Microscope, India) was used to analyze the specimens [2].

### Steatosis scoring

Multiple liver sections stained with hematoxylin and eosin were used to assess the presence of lipid droplets in the hepatocytes; the results are presented as a percentage of the fat content [38, 39].

### Sperm parameters

The sperm count and morphology were assessed according to WHO guidelines [40]. The caudal epididymis was collected, placed in 1 mL of PBS buffer and crushed with a Stiletto blade, and an aliquot was used for the sperm count. The spermatozoa were counted under a light microscope using a Neubauer counting chamber (Marienfeld, Germany). The same sample was used to produce morphology slides stained with hematoxylin & eosin, and sperm with hook-shaped heads and no deformities of the head, neck, or tail were categorized as normal if not they were classified as abnormal [41, 42].

### Gene expression

The TRIzol (Sigma, Cat # T9424-200ML) procedure was used for complete RNA isolation from liver, adipose and testis tissues. Further, a Thermo Fisher Scientific Nanodrop-2000 spectrophotometer and agarose gel electrophoresis were used to evaluate the purity and integrity of the extracted RNA. Using a cDNA synthesis kit (Bio-Rad Kit, Cat # 1,708,891, USA, Inc.), 1 µg of RNA was converted to cDNA by reverse transcription, and its purity was assessed with a spectrophotometer. To perform RT‒PCR (CFX 96, Bio-Rad, USA), TB Green Premix Ex Taq-II (Takara, Cat # RR820A, Bio, Inc.) was used utilizing the three-step PCR melting curve method. The 2^− ΔΔCT^ approach was used to compute the relative mRNA expression of the genes by normalizing them to β-actin [43, 44]. Using the software Primer 3.0, gene-specific primers were designed (The primer list is given in the supplementary data Table 2).

### Statistical measurements

The data are presented as the means ± standard errors of the means (SEMs) and multiple group comparison analysis was performed using one-way ANOVA for each experiment to determine whether any significant differences were present between the groups as mentioned in figure legends. The statistical analysis was conducted, graphs were prepared using Graph Pad Prism 8, and differences with P values < 0.05 were considered significant.

## Results

### Caloric restriction in high-fat diet-fed Wistar rats was associated with reduced body weight, dysregulated sperm function and DNMT’s gene expression

**Fig. 1:**
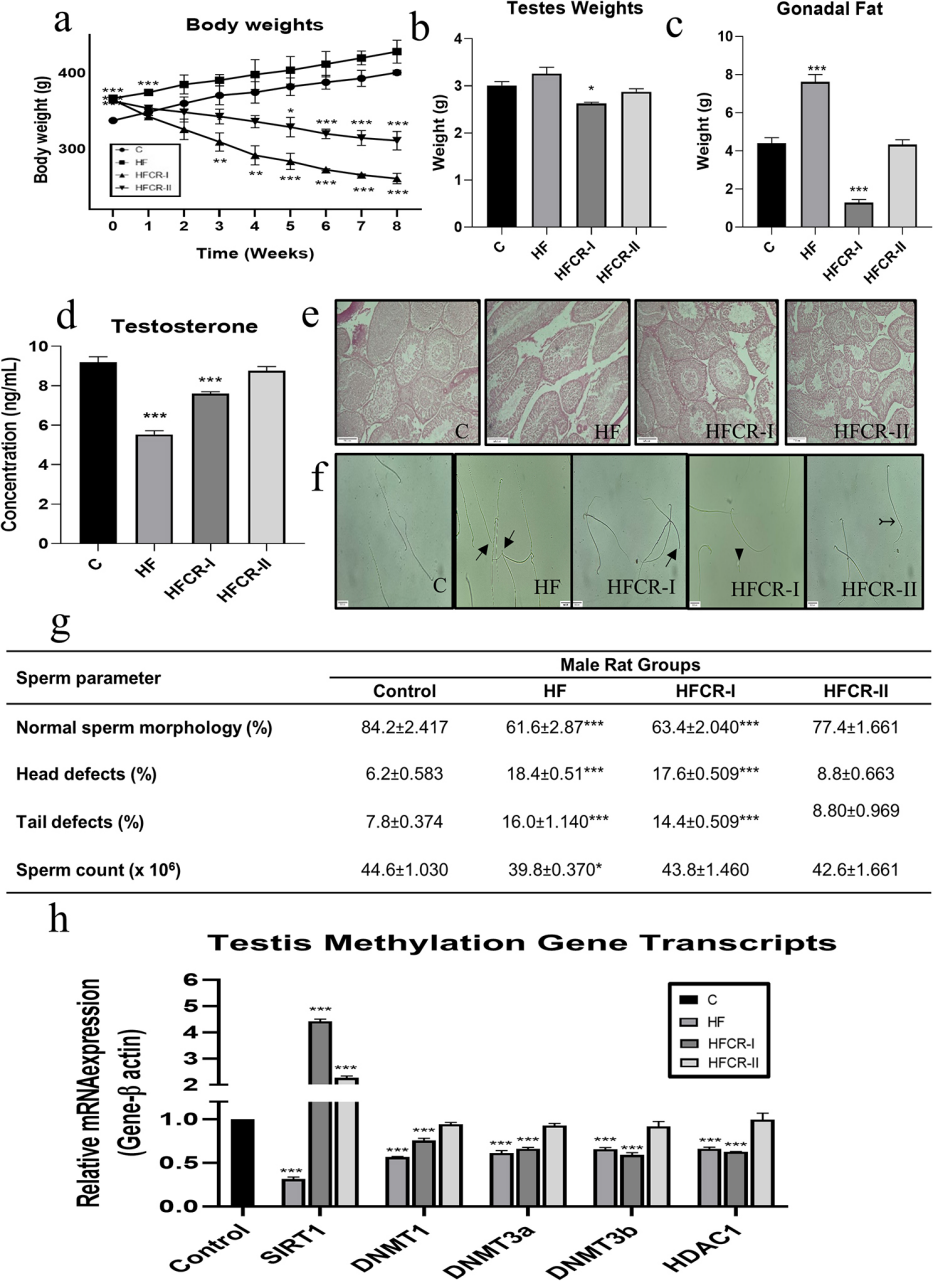
Effect of caloric restriction in high-fat diet-induced obese on fertility of male parents. (**a**) Body weights (**b**) Testes weights. (**c**) Gonadal Fat weights. (**d**) Testosterone levels. (**e**) Representative photomicrograph (40X) of sperm morphology of male parents stained with H & E where➝ triangle headed arrow indicate the decapitation, ▶ pointed triangle indicate seperated head, ↣ inward arrow indicates bent tail. (**f**) Representative photomicrograph (40X) of testis histology of male parents. (**g**) Sperm parameters. (**h**) Testes DNA methylation gene transcripts. Data was presented as mean ± SEM where statistically significance of *P < 0.05; **P < 0.01; ***P < 0.001 vs. controls. All the groups were analysed using one-way ANOVA with Dunnett’s post hoc test

The body weights during calorie restriction phase were increased in the HF male parents and significantly decreased in the HFCR-I & HFCR-II male parents but a prominent decrease was observed in HFCR-I compared to the control male parents (Fig. 1a). At the end of calorie restriction phase, the testis weights were increased in HF, significantly decreased HFCR-I and comparable in HFCR-II male parents to their controls (Fig. 1b). In addition, the gonadal fat weights were significantly increased in the HF, significantly decreased in the HFCR-I and comparable in the HFCR-II male parents to the controls (Fig. 1c). The plasma testosterone levels were significantly decreased in the HF & HFCR-I and comparable in the HFCR-II male parents to the controls (Fig. 1d). Further, the testes histopathology results indicated that only the HF male parents exhibited reduced spermatogenesis (Fig. 1e). The morphological abnormalities of spermatozoa, such as decapitation and bent tails, were significant in the HF & HFCR-I male parents than in the controls. The sperm count was significantly decreased in the HF and comparable in the HFCR-I & HFCR-II male parents to the controls (Fig. 1f & g). The mating frequency was less in HF & HFCR-I males, where some animals failed to reproduce based on observation of the vaginal copulation plugs formation and successful offspring production than the HFCR-II and control males (Supplementary Table 3). However, among successfully mated animals, the litter size remained unchanged, but there was an altered sex ratio with significantly fewer female births to the HFCR-I males than the control males (Supplementary Table 4). In the testes, the gene expression of SIRT1 was significantly downregulated in the HF males and significantly upregulated in the HFCR-I & HFCR-II males compared to the controls. Further, the mRNA expression profiles of DNMT1, DNMT3a and DNMT3b were significantly lower in HF & HFCR-I males than in the controls. The gene expression of HDAC1 was significantly lower in the HF & HFCR-I males than in controls (Fig. 1 h).

### Preconceptional paternal caloric restriction in high-fat diet-fed Wistar rats demonstrated attenuation of energy homeostasis in F1 offspring via the AMPK-SIRT1 pathway

**Fig. 2:**
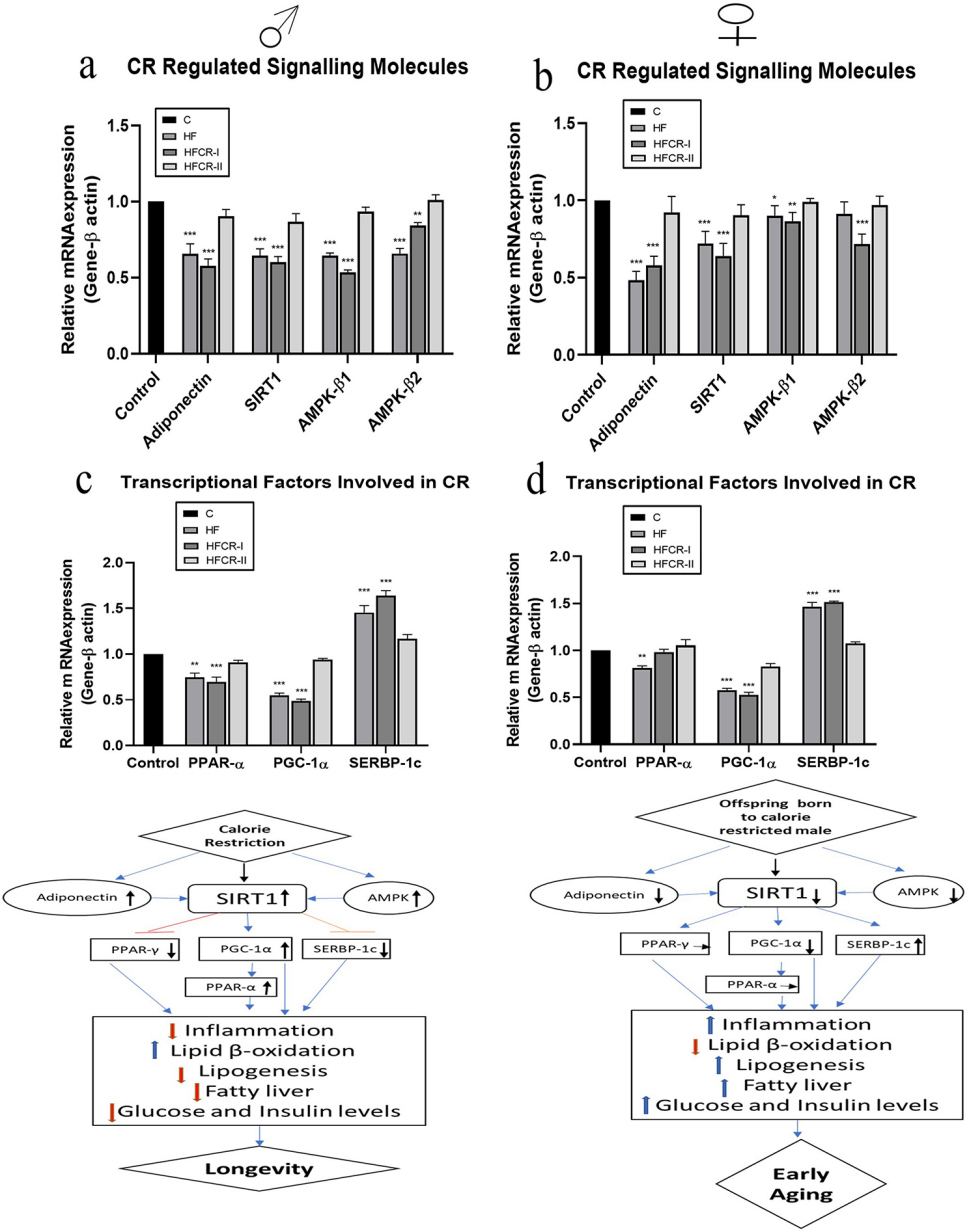
Effect of paternal caloric restriction on offspring signaling molecules. (**a**) Gene expression of energy metabolism pathways in the male offspring. (**b**) Gene expression of energy metabolism pathways in the female offspring. (**c**) Gene expression of the transcriptional factors involved in energy metabolism in the male offspring. (**d**) Gene expression of transcriptional factors involved in energy metabolism in the female offspring. Data was presented as mean ± SEM where statistically significance of *P < 0.05; **P < 0.01; ***P < 0.001 vs. controls. All the groups were analysed using one-way ANOVA with Dunnett’s post hoc test

The expression of adiponectin gene in adipose tissue significantly downregulated in the (both male and female) offspring of HF & HFCR-I when compared to the respective age- and gender-matched controls (Fig. 2a & b). The expression of the SIRT1 gene in the liver was significantly downregulated in the offspring of HF & HFCR-I than their respective controls (Fig. 2a & b). The liver AMPK-β1 gene expression significantly decreased in the offspring of HF & HFCR-I and AMPK-β2 gene expression significantly decreased in the male offspring of HF & HFCR-I and female offspring of HFCR-I only compared to their respective controls (Fig. 2a & b).

In the liver, the gene expression of PPARα was significantly decreased in HF & HFCR-I male offspring whereas in female offspring, it was significantly decreased in the HF and comparable in the HFCR-I & HFCR-II offspring compared to the respective controls (Fig. 2c & d). The PGC-1α expression was significantly decreased in all the offspring of HF & HFCR-I groups and was comparable in HFCR-II group. Further, the SERBP-1c expression was significantly increased in all the offspring of HF & HFCR-I groups and was comparable in the HFCR-II group compared to the controls (Fig. 2c & d). The gene expression of leptin and PPAR-γ was also analyzed but was found to be comparable among all the offspring (data not presented).

### Preconceptional paternal caloric restriction in diet-induced obese Wistar rats led to increased postweaning body weight gain and body fat percentage in F1 offspring

**Fig. 3:**
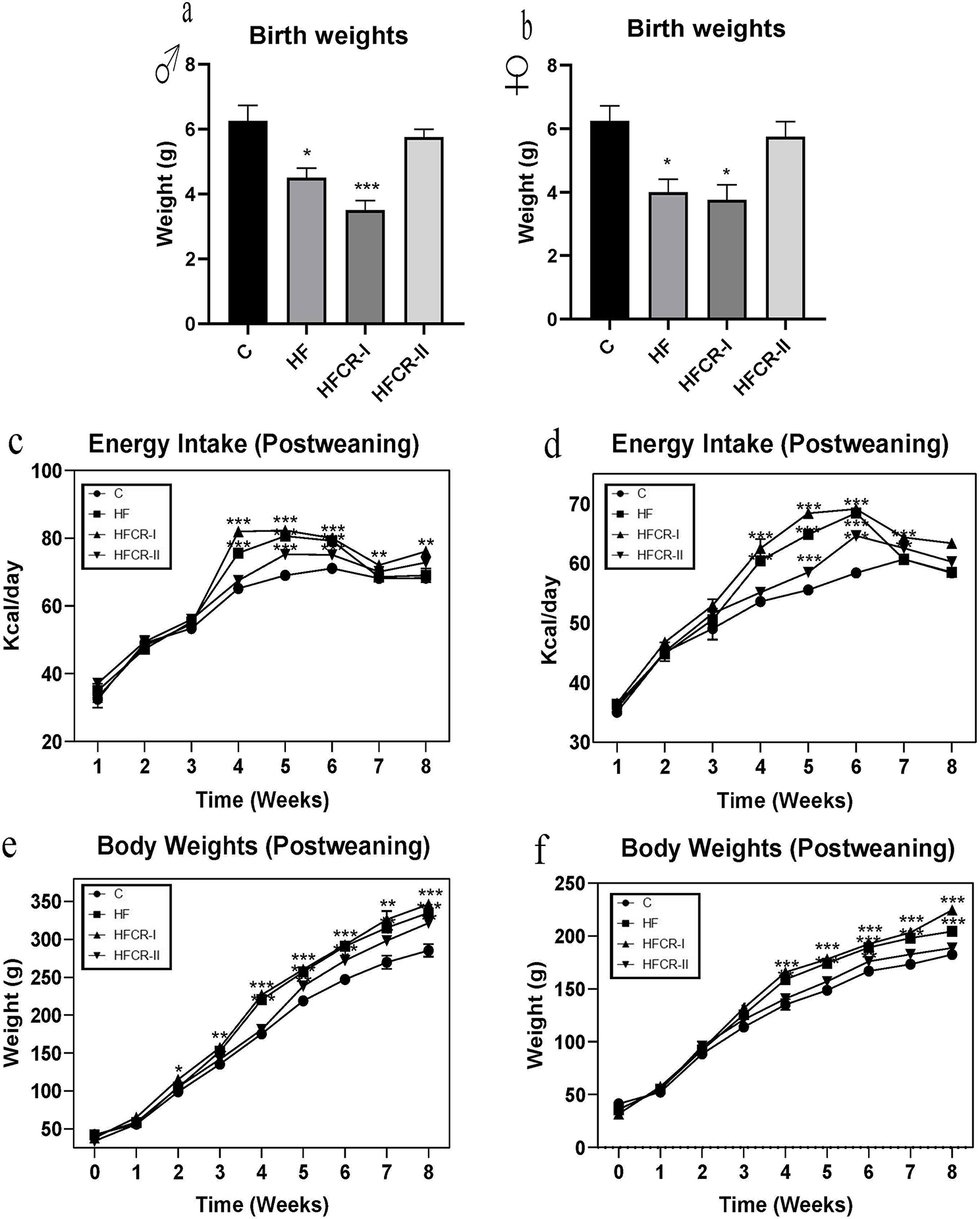
Effect of paternal caloric restriction on offsprings body weights . (**a**) Birth weights of male offspring. (**b**) Birth weights of female offspring. (**c**) Postweaning energy intake in the male offspring. (**d**) Postweaning energy intake in the female offspring. (**e**) Postweaning body weights in the male offspring. (**f**) Postweaning body weights in the female offspring. Data was presented as mean ± SEM where statistically significance of *P < 0.05; **P < 0.01; ***P < 0.001 vs. controls. All the groups were analysed using one-way ANOVA with Dunnett’s post hoc test

**Fig. 4:**
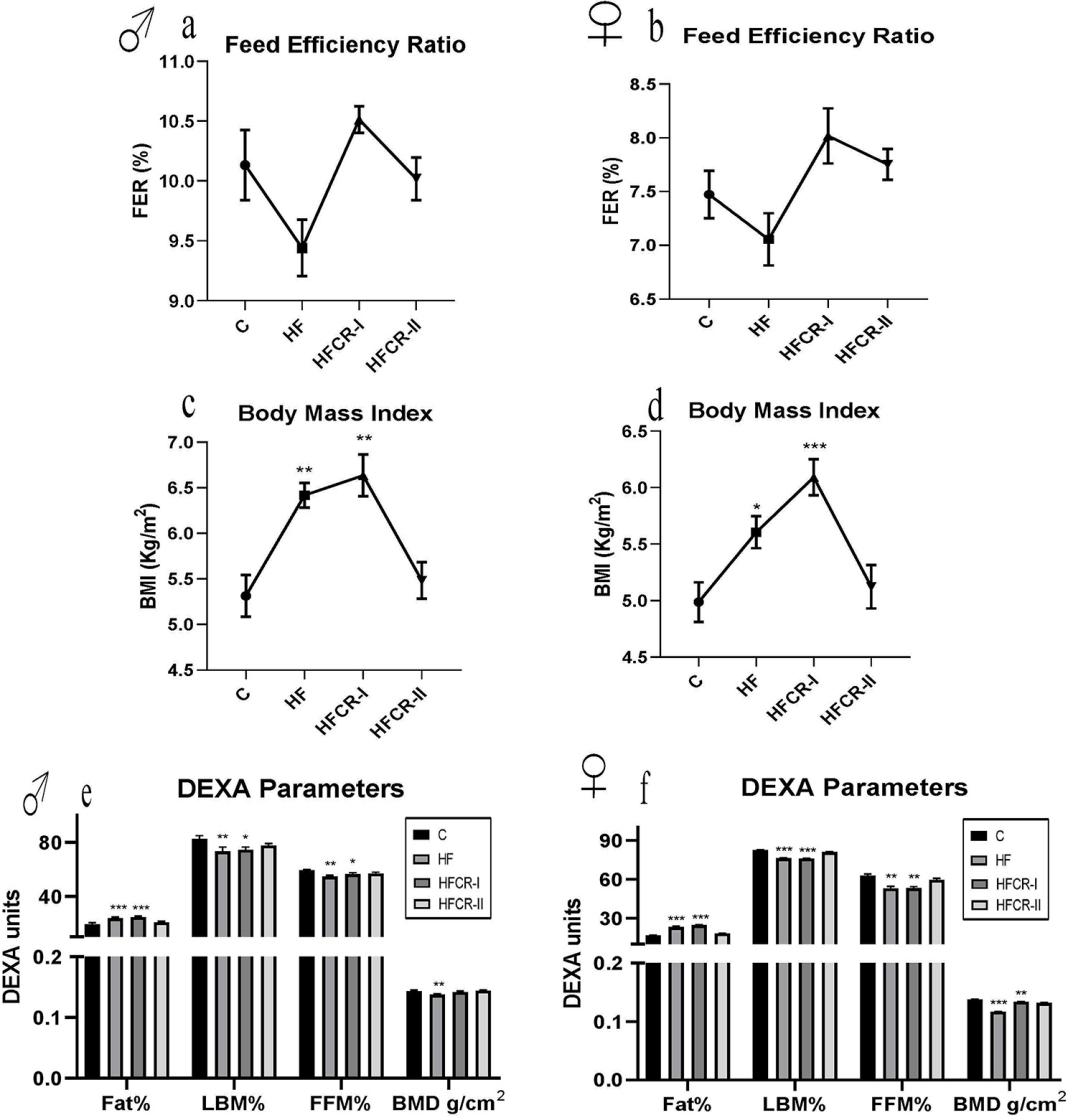
Effect of paternal caloric restriction on offsprings anthropometric measures. (**a**) FER of male offspring. (**b**) FER of female offspring. (**c**) BMI of male offspring. (**d**) BMI of female offspring. (**e**) DEXA measurements of the male offspring. **(f**) DEXA measurements of the female offspring. Data was presented as mean ± SEM where statistically significance of *P < 0.05; **P < 0.01; ***P < 0.001 vs. controls, All the groups were analysed using one-way ANOVA with Dunnett’s post hoc test

The birth weights of the offspring (both males and females) of the HF & HFCR-I groups were significantly lower than those of the control group (Fig. 3a & b). However, the offspring of the HF & HFCR-I groups showed a significant increase in body weights followed by HFCR-II after weaning, which was in line with their energy intake (Fig. 3c-f). FER of the offspring was considerably lower in the HF and higher in the HFCR-I than the HFCR-II group compared to the control group (Fig. 4a & b). In consistent, the offspring of HFCR-I along with the HF group showed significantly increased BMI compared to the control group offspring (Fig. 4c & d). The DEXA results have demonstrated that the total body fat significantly increased in the offspring of the HF & HFCR-I groups and the LBM% & FFM% significantly decreased in the offspring of the HF & HFCR-I groups compared to their controls. The BMD was significantly lower in male and female HF offspring and only female HFCR-I offspring than the respective controls (Fig. 4e & f). Further, the organ weights of HF & HFCR-I offspring have non-significantly increased except liver weights significantly increased in male and female offspring and kidney weights significantly increased in HFCR-I female offspring (Supplementary Table 5a & b). The male offspring of HF & HFCR-I groups significantly showed an increased AI, whereas, among the female offspring, only the HFCR-I group showed a significant increase in the AI compared to their controls. However, the HFCR-II group showed comparable AI with the control group (Supplementary Table 5a & b).

### Preconceptional paternal caloric restriction in diet-induced obese Wistar rats was associated with the progression of proinflammatory status and oxidative stress in F1 offspring

The plasma levels of adipocytokines such as adiponectin were found to be comparable in all the offspring among all the groups; whereas leptin levels were found to be significantly elevated in the male and female offspring of the HF & HFCR-I groups compared to their respective age- and gender-matched controls (Table 1a & b). The circulatory levels of proinflammatory cytokines, such as IL-1β, IL-6, MCP-1 and TNF-α, were significantly increased in male and female offspring of the HF, HFCR-I and HFCR-II groups than their respective controls. The levels of proinflammatory cytokines were significantly higher in the HF and HFCR-I groups than in the HFCR-II group (Table 1a & b). Further, the plasma levels of oxidative stress markers, such as TAC and catalase activity were significantly decreased and increased respectively in the male and female offspring of HF, HFCR-I and HFCR-II groups compared to controls and also respective significant differences were observed in HF and HFCR-I compared to HFCR-II (Table 1a & b).

**Table 1:** Inflammatory markers

	C	HF	HFCR-I	HFCR-II
**(a) (Plasma concentration)**	**Male offspring**			
Adiponectin (ng/mL)	0.453 ± 0.02	0.467 ± 0.01	0.460 ± 0.01	0.480 ± 0.04
Leptin (ng/mL)	16.2 ± 0.38^a^	20.29 ± 0.22^b^	21.44 ± 0.45^b^	17.6 ± 0.31^a^
IL-1β (pg/mL)	33.98 ± 1.91^a^	65.53 ± 1.45^b^	68.50 ± 1.05^b^	52.85 ± 1.49^c^
IL-6 (pg/mL)	463.2 ± 1.49^a^	685.0 ± 2.58^b^	654.6 ± 1.11^c^	597.3 ± 0.55^d^
MCP-1 (pg/mL)	554.9 ± 1.88^a^	716.2 ± 1.71^b^	733.3 ± 1.66^c^	695.8 ± 1.77^d^
TNF-α (pg/mL)	4.710 ± 0.23^a^	5.928 ± 0.03^b^	7.010 ± 0.10^c^	5.643 ± 0.17^b^
TAC (µM)	282.7 ± 4.27^a^	240.7 ± 2.53^b^	229.2 ± 3.33^b^	256.9 ± 1.44^c^
Catalase Activity (U/L)	0.529 ± 0.33^a^	0.834 ± 0.02^b^	1.106 ± 0.07^c^	0.716 ± 0.01^d^
**(b) (Plasma concentration)**	**Female offspring**			
Adiponectin (ng/mL)	0.493 ± 0.02	0.453 ± 0.02	0.463 ± 0.01	0.433 ± 0.03
Leptin (ng/mL)	15.3 ± 0.37^a^	20.2 ± 0.24^b^	22.3 ± 0.18^c^	16.2 ± 0.17^a^
IL-1β (pg/mL)	47.0 ± 1.1^a^	120.9 ± 2.8^b^	123.4 ± 1.5^b^	98.27 ± 1.5^c^
IL-6 (pg/mL)	401.4 ± 3.96^a^	695.8 ± 1.41^b^	654.9 ± 1.2^c^	608.8 ± 1.97^d^
MCP-1 (pg/mL)	401 ± 1.05^a^	596.9 ± 1.23^b^	611.5 ± 3.15^c^	563.2 ± 1.91^d^
TNF-α (pg/mL)	2.76 ± 0.11^a^	5.26 ± 0.23^b^	7.92 ± 0.09^c^	4.38 ± 0.30^b^
TAC (µM)	265.3 ± 4.49^a^	237.3 ± 4.97^b^	203.2 ± 3.43^c^	244.0 ± 2.64^b^
Catalase Activity (U/L)	0.795 ± 0.01^a^	0.992 ± 0.00^b^	1.074 ± 0.07^b^	0.937 ± 0.01^ab^

Data was presented as mean ± SEM where values with unlike superscript letters were significantly different *p* < 0.05. The groups were analysed using one-way ANOVA with Tukey’s post hoc test.

### Preconceptional paternal caloric restriction in diet-induced obese Wistar rats resulted in altered lipid metabolism in F1 offspring

**Fig. 5:**
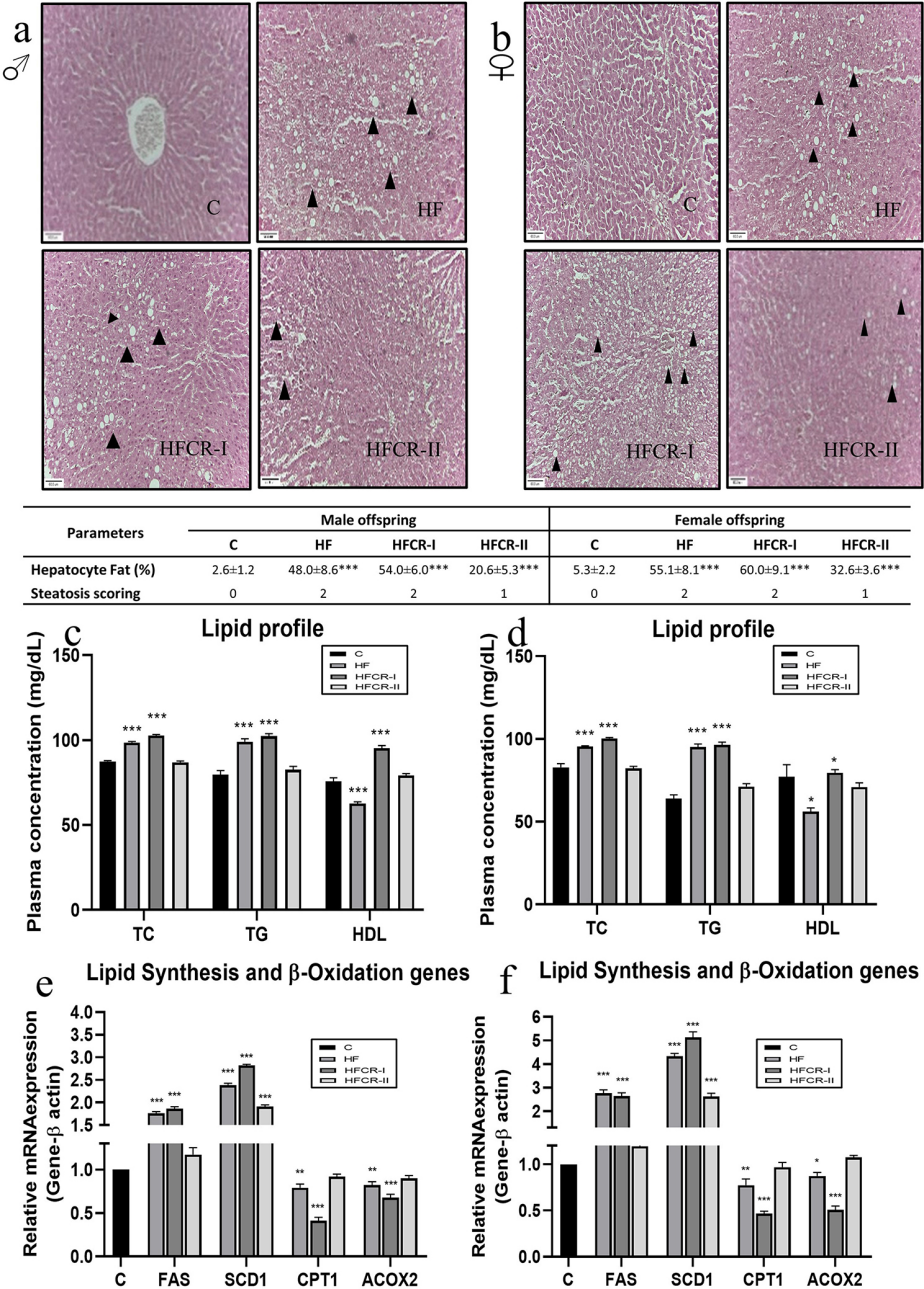
Effect of paternal caloric restriction on offsprings lipid metabolism. (**a & b**) Representative photomicrographs (40X) of liver histology of of male and female offspring respectively where ▶ pointed triangle indicate presence of lipid droplets. (**c**) Lipid profiles of the male offspring. (**d**) Lipid profiles of the female offspring. (**e**) Lipogenesis and lipid oxidation gene expression in the male offspring. (**f**) Lipogenesis and lipid oxidation gene expression in the female offspring. Data was presented as mean ± SEM where statistically significance of *P < 0.05; **P < 0.01; ***P < 0.001 vs. controls. All the groups were analysed using one-way ANOVA with Dunnett’s post hoc test

The liver histopathology studies have shown, that the male and female offspring of the HF & HFCR-I groups developed moderate steatosis, whereas those in the HFCR-II group developed mild steatosis according to the steatosis scoring by Kleiner et al. (Figure. 5a & b). Increased hepatocyte fat% was observed in female offspring of HF, HFCR-I and HFCR-II groups than male offspring but a significant increase was only observed in HFCR-II female than male offspring (Supplementary Table 6). The lipid profile results of the offspring showed that the plasma levels of total cholesterol & triglycerides were significantly elevated in the HF & HFCR-I groups, whereas the HDL cholesterol levels were significantly reduced in the HF group and significantly elevated in the HFCR-I group than the respective controls (Fig. 5c & d). Further, investigated the gene expression profiles of rate-limiting enzymes involved in lipid metabolism of the offspring. The genes involved in lipogenesis, FAS was significantly upregulated in the offspring of HF & HFCR-I groups compared to their controls & also significantly upregulated in females than males and SCD1 was significantly upregulated in the offspring of HF, HFCR-I & HFCR-II groups compared to their controls & also significantly upregulated in females than males (Fig. 5e & f) (Supplementary Table 6). However, the β-oxidation of fatty acid genes, i.e., ACOX2 & CPT1 were significantly downregulated in the HF & HFCR-I offspring and comparable in the HFCR-II offspring compared to their respective controls (Fig. 5e & f). ACOX2 was also significantly downregulated in HFCR-I and HFCR-II females than males (Supplementary Table 6).

### Preconceptional paternal caloric restriction in diet-induced obese Wistar rats led to impaired glucose metabolism and insulin sensitivity in F1 offspring

**Fig. 6:**
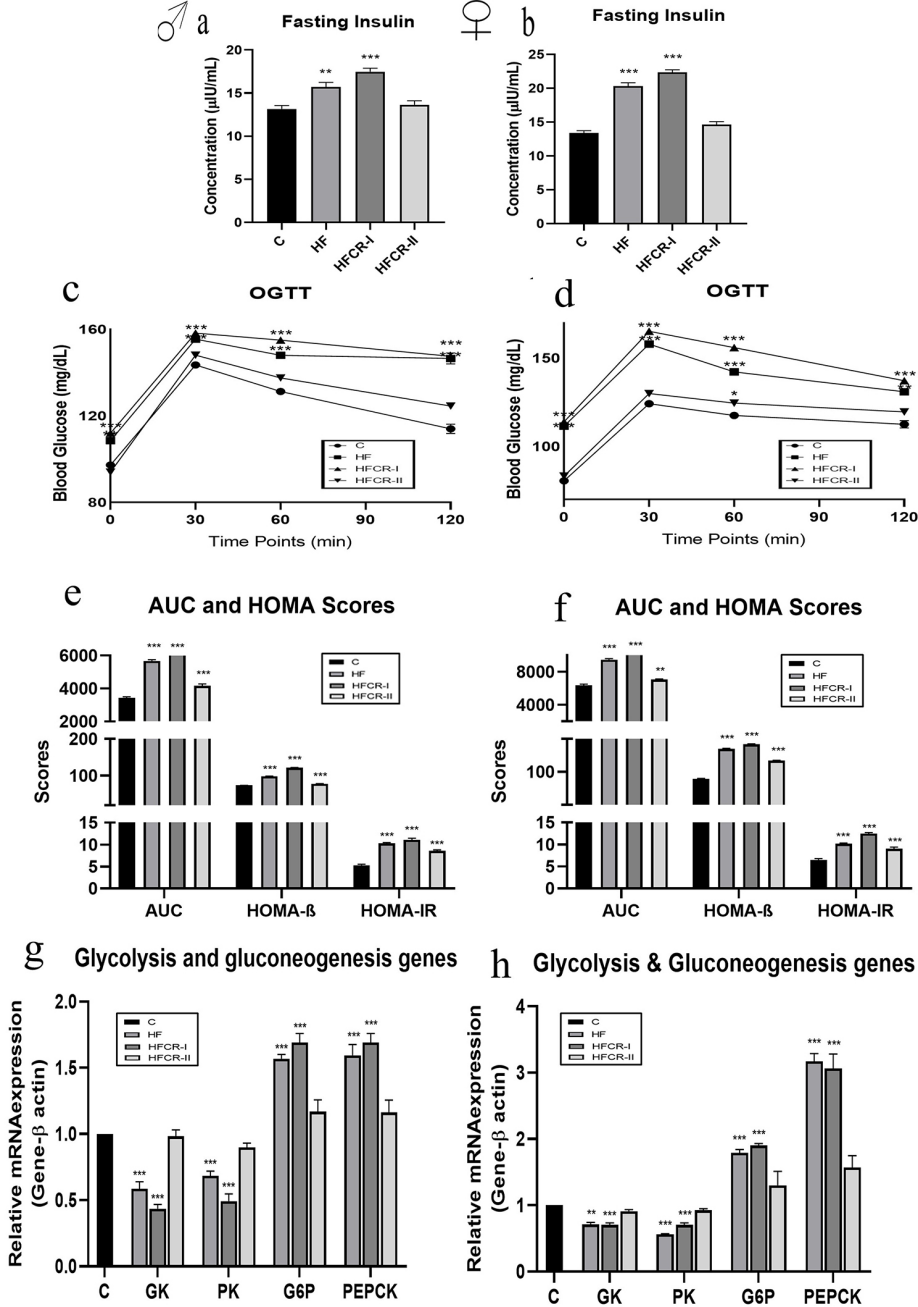
Effect of paternal caloric restriction on offsprings insulin sensitivity. (**a**) Fasting insulin of male offspring. (**b**) Fasting insulin of female offspring. (**c**) OGTT of male offspring. (**d**) OGTT of female offspring. (**e**) AUC & HOMA scores of male offspring. (**f**) AUC & HOMA scores of female offspring. (**g**) Glycolysis & gluconeogenesis enzymes gene expression in the male offspring. (**h**) Glycolysis enzymes & gluconeogenesis gene expression in the female offspring. Data was presented as mean ± SEM where statistically significance of *P < 0.05; **P < 0.01; ***P < 0.001 vs. controls. All the groups were analysed using one-way ANOVA with Dunnett’s post hoc test

The circulatory levels of fasting insulin were significantly elevated in both male and female offspring of the HF & HFCR-I groups compared to their respective controls (Fig. 6a & b) and also significantly elevated in female than male offspring (Supplementary Table 6). The OGTT results of offspring have demonstrated that the HF & HFCR-I groups showed significantly increased fasting glucose levels and delayed glucose clearance from the circulation compared to the controls (Fig. 6c & d). Similarly, the AUC glucose, HOMA-IR & HOMA-β scores were significantly higher in the offspring of the HF & HFCR-I groups than the HFCR-II group compared to controls (Fig. 6e & f). In addition, gene expression studies in liver tissue revealed that the glycolytic enzyme-encoding genes GK and PK were significantly downregulated in male and female offspring of the HF & HFCR-I groups compared to their respective controls (Fig. 6g & h) and also significantly downregulated in HFCR-I females than males (Supplementary Table 6). The liver gluconeogenesis enzyme-encoding genes G6-P and PEPCK were significantly upregulated in both male and female offspring of the HF & HFCR-I groups compared to their respective controls (Fig. 6g & h) but PEPCK has also significantly upregulated in female than male offspring (Supplementary Table 6).

## Discussion

The present study emphasizes the impact of preconceptional paternal calorie restriction (CR) on various metabolic and physiological parameters in high-fat-fed male Wistar rats and their F_1_ offspring. The findings shed light on the intricate interplay between paternal diet, metabolic health, and offspring outcomes, highlighting potential mechanisms underlying transgenerational metabolic programming.

Under the influence of the HF diet, the male parents demonstrated increased body weights, gonadal fat weights and reduced testosterone levels which likely suggest their compromised male fertility [45–47]. It was also observed that the abnormal testis & sperm morphologies in the HF group led to reduced spermatogenesis and reproduction capability which is in accordance with the assessment of male fertility [40]. Studies have reported that paternal undernutrition reduces male fertility which in turn is responsible for metabolic disorders in the offspring [13, 26, 27]. In the present study, a noteworthy observation emerged in the group subjected to a 50% CR (HFCR-I) within the diet-induced obese Wistar rats. Instead of rectifying the detrimental effects induced by the high-fat diet, this level of CR resulted in a state akin to undernutrition. Consequently, this undernourished condition had adverse repercussions on male fertility and impacted the health of their offspring. Whereas, implementing a 40% CR in diet-induced obese Wistar rats (HFCR-II) nearly reinstated the effects induced by a high-fat diet. Current findings also noticed altered DNMT gene expression in the testes of parents that may show epigenetic influence on their offspring metabolism. The offspring of HF and HFCR-I have shown obesogenic tendencies with increased inflammation leading to impaired glucose homeostasis by exhibiting different pathophysiology.

A study has found that moderate CR of obese male mice leads to the upregulation of SIRT1 which has beneficial effects on reproductive health [48]. In line, another study also demonstrated that SIRT1 knock-out mice has resulted in altered number & morphology of spermatozoa, causing reduced fertility [49]. Similar trends were noticed in the present study, where the SIRT1 downregulation in HF male parents is compromising their fertility. Moreover, in HFCR-I, despite SIRT1 is upregulated, their fertility was compromised due to nutrient deprivation caused by severe CR. These findings likely suggest that HF and HFCR-I may have different molecular mechanisms impacting male fertility. Further in both the cases, it was proven to have reduced mating frequency. The HFCR-I did not alter the litter size but did alter the male-to-female sex ratio which is commensurate with a study [50]. This shows that HFCR-I males differentially produce sperms bearing X and Y chromosomes, hence the birth of male offspring is favored.

SIRT1, a histone deacetylase, causes the silencing of genes by recruiting DNMTs, and it was also reported that SIRT1 downregulation leads to the downregulation of DNMT1, DNMT3a, and DNMT3b in the breast cancer cells of mice [51, 52]. SIRT1 is also known to inhibit the other histone deacetylase HDAC1 [53]. In the present study, irrespective of decreased and increased SIRT1 expression in HF and HFCR-I respectively; there was a downregulation of DNMT1, DNMT3a, DNMT3b and HDAC1 which could have modulated the methylation/imprinting of genes in the offspring affecting their health. Thus, this establishes the relation between the paternal CR to their DNMT’s differential expression affecting the metabolism in their offspring.

While there is ample research available on how maternal nutritional interventions affect metabolic pathways in offspring, the effects of paternal nutritional strategies remain relatively underreported [15]. In rats, maternal CR downregulates the AMPK pathway in offspring, which subsequently causes various metabolic disorders, such as obesity, CVD, and steatohepatitis. Dysregulation of the underlying molecular mechanism has been linked to hyperacetylation of PGC-1α and reduced SIRT1 expression and function [54, 55]. Similarly, in the present study, the male and female offspring born to HF and HFCR-I have shown the downregulation of AMPK/SIRT1/adiponectin pathways that led to the dysregulation of inflammation, lipid and glucose metabolism in them which clearly demonstrates the importance of paternal diet. This also indicates the effects of paternal CR are mostly similar to the maternal CR. In HFCR-II offspring the gene expression was mostly comparable to the controls. Hence, moderate CR can be considered as an effective weight loss regime in obese individuals without causing much effect on their future generations.

Earlier mice studies reported that the birthweights of male offspring are reduced under a paternal high-fat diet [56], and it was also reported that paternal malnutrition led to a decreased birth weight of female offspring [25]. Our results are in accordance with these studies in which low birthweights of male and female offspring of HF and HFCR-I showed that these offspring may be prone to metabolic disorders at an early age. It was reported earlier in mice that fathers’ high-fat diet exposure leads to increased body weight gain in offspring [57], and paternal malnutrition leads to weight gain in offspring at an early age [25, 27]. Additionally, in the present study, male and female offspring of HF and HFCR-I gained more weight after weaning, which goes along with their energy intake. This shows the pattern of premature births or children born small catching up weight at an early age. Leptin resistance with increased FER leads to obesity [58]. Further, the increased plasma leptin in the HF and HFCR-I offspring shows that they are prone to leptin resistance irrespective of their FER differences, indicating that they exhibit different pathophysiology of obesity. In mice, the father’s high-fat diet led to increased fat mass in the offspring [57], and the paternal low-protein diet in rats was also associated with increased fat mass and organ weights in the offspring [26, 59]. The present findings are also in line with those of previous studies in which the total body fat percentage increased with increased adiposity index in HF and HFCR-I offspring, demonstrating that they exhibit imbalanced body fat accumulation.

High-fat diet exposure in fathers leads to increased serum lipid and lipid synthesis in offspring [19, 60, 61]. In mice, a paternal low-protein diet has also shown increased total cholesterol and triglycerides in the offspring [59] and increased lipogenesis gene expression in male offspring [62]. The present study revealed greater plasma lipid levels in HF and HFCR-I offspring than in HFCR-II offspring, suggesting that a paternal high-fat diet and CR lead to obesogenic effects in offspring, which was further confirmed by their increased mRNA expression of the lipid synthesis genes FAS and SCD1 and decreased expression of the lipid β-oxidation genes CPT1 and ACOX2. The expression difference was greater in females than in males, demonstrating more adverse sex-specific effects of paternal diet. Interestingly, our results showed that a paternal high-fat diet and 50% CR increased liver weight and steatosis in the offspring, and these effects were severe in female offspring. The increased total cholesterol and triglycerides and the deposition of fat droplets in hepatocytes resulted in fatty liver conditions and dyslipidemia in the HF and HFCR-I offspring. These results illustrate that a father’s obesity can be inherited to their offspring, while paternal CR prompts the body to adapt to an energy-conserving mode in anticipation of limited resources. This adaptation may have led to reprogramming and imprinting in the sperm, potentially influencing the metabolic health of subsequent generations.

Multiple studies on paternal high-fat diets have shown increased inflammation in offspring [22, 60, 63, 64]. A paternal low-protein diet has increased the TNF-α levels in the offspring [27]. The levels of the proinflammatory cytokines IL-6, IL-1β, TNF-α and MCP-1 in the circulation are greater in the HF and HFCR-I groups than in the HFCR-II group, resulting in increased inflammation, which is further supported by increased leptin and decreased TAC and catalase activity. In Wistar rats fed high-caloric diets, hypoadiponectinemia and hyperleptinemia restrict energy expenditure and glucose consumption, leading to reduced glycolysis and fatty acid metabolism [2]. A preconceptional father’s high-fat diet alters insulin sensitivity and glucose metabolism [60, 61]. The paternal low-protein diet has also led to impaired blood glucose levels in both male and female offspring [26, 27]. The elevated levels of fasting insulin and glucose, along with the delayed clearance of glucose upon oral glucose challenge in offspring from the HF and HFCR-I corroborate their β-cell dysfunction and dysregulated glucose homeostasis. This finding was further supported by the increased AUC, HOMA-IR and HOMA-β with decreased and increased expression of glycolytic and gluconeogenesis enzymes respectively. Further female offspring have shown an adverse effect on glucose metabolism genes than male offspring evidencing the sex-specific effects of the paternal diet. These findings demonstrated that the HF and HFCR-I offspring groups exhibited increased inflammation and adiposity, leading to insulin resistance.

Hence this study illustrates that the modulation of AMPK/SIRT1 pathways in the offspring alters inflammation, lipid & glucose metabolism developing obesity and its associated co-morbidities at an early age.

## Conclusion

The findings from the present study show that subjecting high-fat diet-induced obese males to severe caloric restriction (50% CR) results in numerous deleterious effects on the metabolism of their offspring. Conversely, moderate caloric restriction (40% CR) has been shown to mostly reverse the impact of high-fat diet-induced complications on metabolism in both fathers and offspring. These findings highlight the significant influence of paternal health and dietary conditions on subsequent generations. Despite the recognition of the importance of paternal effects, there is limited knowledge on strategies to mitigate them. Future studies should aim to fill this gap by providing fathers with tailored dietary recommendations to optimize the health of their offspring.

### Electronic supplementary material

Below is the link to the electronic supplementary material.

Supplementary Material 1

Supplementary Material 2

Supplementary Material 3

## Data Availability

No datasets were generated or analysed during the current study.

## Abbreviations

HF: High-fat
HFCR-I: High-fat fed rats subjected to 50% caloric restriction
HFCR-II: High-fat fed rats subjected to 40% caloric restriction
SIRT1: Sirtuin1
AMPK: AMP-activated protein kinase
PGC1α: Peroxisome proliferator activated receptor gamma coactivator 1alpha
PPARα: Peroxisome proliferator activated receptor alpha
PPARγ: Peroxisome proliferator activated receptor gamma
SERBP1c: Sterol regulatory element binding protein 1c
SETD2: SET domain containing 2
TM6SF2: Transmembrane 6 superfamily member 2
ATF7: AMP-dependent transcription factor 7
FER: Feed efficiency ratio
BMI: Body mass index
DEXA: Dual X-ray absorptiometry
LBM%: Lean body mass percent
FFM%: Fat free mass percent
WAT: White adipose tissue
AI: Adiposity index
HOMA-β: Homeostasis model assessment of β-cell function
HOMA-IR: Homeostasis model assessment of insulin resistance
OGTT: Oral glucose tolerance test
AUC: Area under curve
BMD: Bone mineral density
TAC: Total antioxidant capacity
DNMT’s: DNA methyl transferases
HDAC1: Histone deacetylase 1
FAS: Fatty acid synthase
SCD1: Stearoyl CoA desaturase 1
ACOX2: Acyl-CoA oxidase 2
CPT1: Carnitine palmitoyltransferase 1
GK: Glucokinase
PK: Pyruvate Kinase
G6P: Glucose 6-phosphatase
PEPCK: Phosphoenolpyruvate carboxykinase
