# Supplementary figures and images for: Preconceptional paternal caloric restriction of high-fat diet-induced obesity in Wistar rats dysregulates the metabolism of their offspring via AMPK/SIRT1 pathway
Source: Lipids Health Dis. 2024 Jun 8;23:174. doi: 10.1186/s12944-024-02161-6 (PMC11162063; doi:10.1186/s12944-024-02161-6)

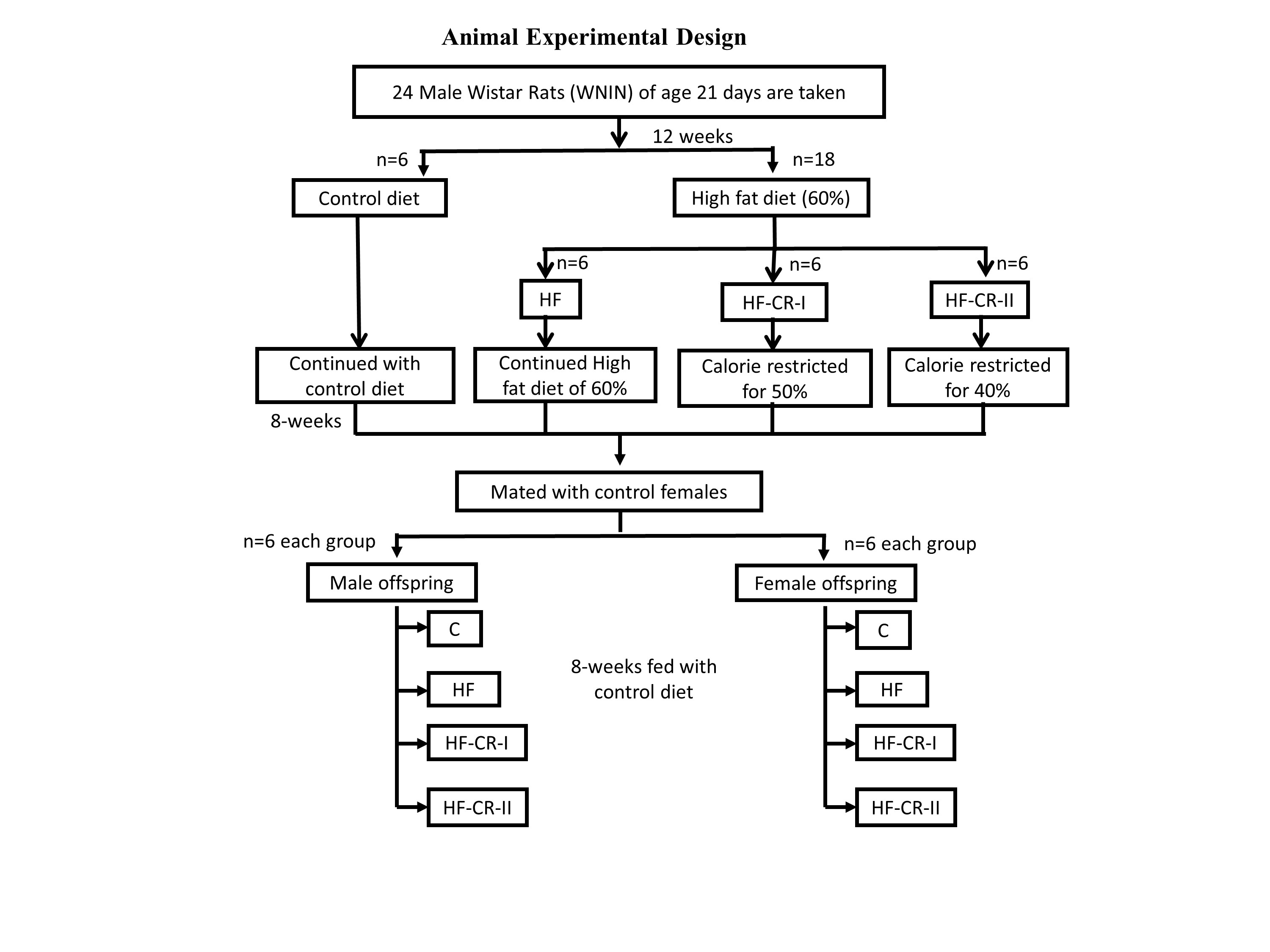

Supplement: Supplementary file 1 — Supplementary Material 1 [file 12944_2024_2161_MOESM1_ESM.jpg]

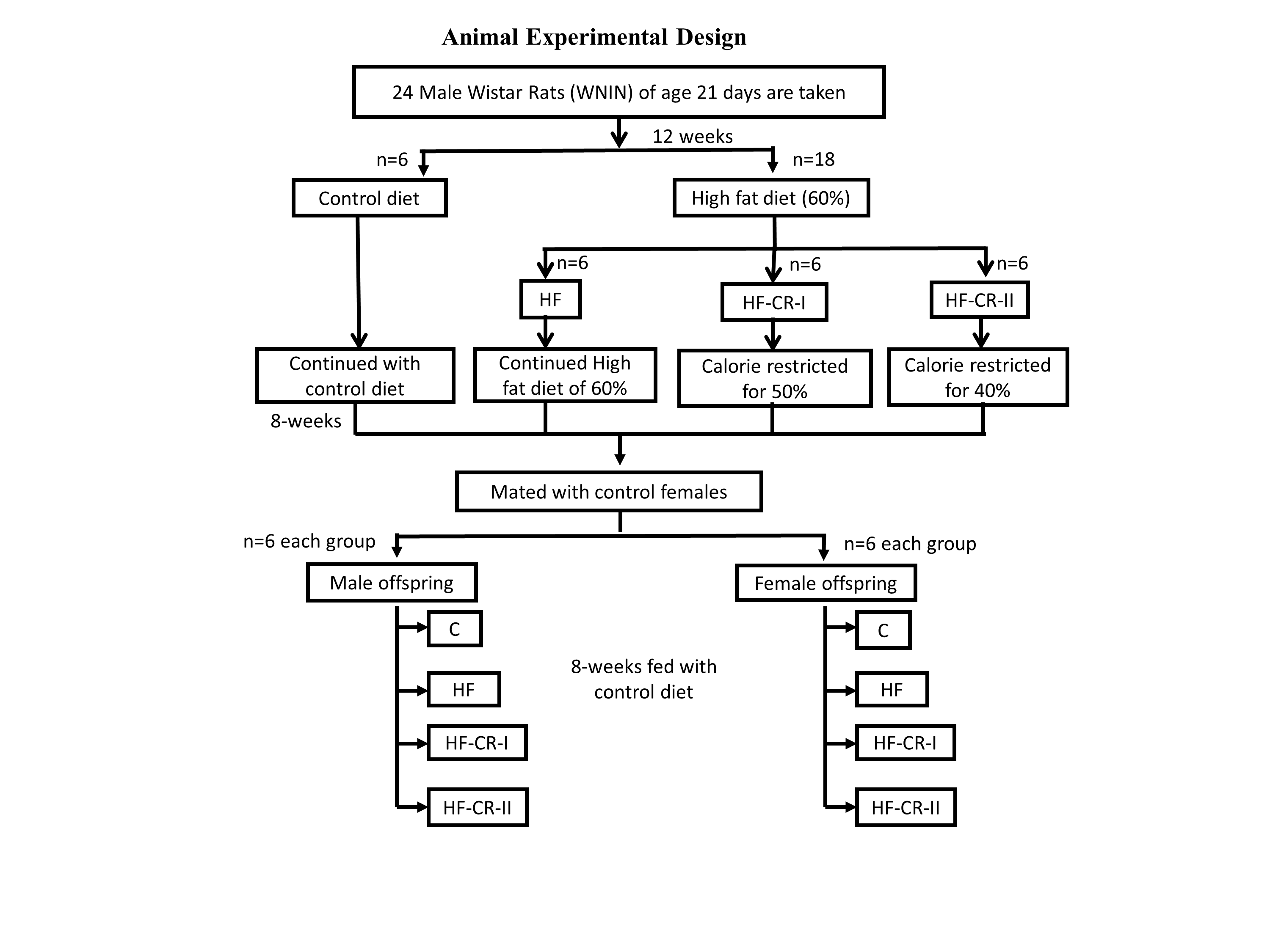

Supplement: Supplementary file 2 — Supplementary Material 2 [file 12944_2024_2161_MOESM2_ESM.tif]
