# Supplementary material for: Preconceptional paternal caloric restriction of high-fat diet-induced obesity in Wistar rats dysregulates the metabolism of their offspring via AMPK/SIRT1 pathway
Source: Lipids Health Dis. 2024 Jun 8;23:174. doi: 10.1186/s12944-024-02161-6 (PMC11162063; doi:10.1186/s12944-024-02161-6)
Supplement: Supplementary file 3 — Supplementary Material 3 [file 12944_2024_2161_MOESM3_ESM.docx]

**Supplementary Tables.**

**Table. 1 Composition of Control and High fat diet (60%).**

| **Ingredients** | **g/kg of diet** | |
| --- | --- | --- |
|  | **Control** | **High fat (60%)** |
| Corn starch | 397.486 | 230 |
| Maltodextrin | 132 | - |
| Sucrose | 100 | 40 |
| Casein | 200 | 270 |
| Soybean oil (without additives) | 70 | - |
| Lard | - | 360 |
| Cellulose | 50 | 50 |
| AIN 93 G Mineral mix | 35 | 35 |
| AIN 93 G Vitamin mix | 10 | 10 |
| L-cysteine | 3 | 3 |
| Choline bitartarate (41.1% choline) | 2.5 | 2.5 |

Energy level of the control diet is 3.95Kcal/g where Carbohydrate ratio is 60-64%, Protein ratio is 20% and Fat ratio is 16%.

Energy levels of the high fat diet is 5.4Kcal/g where Carbohydrate ratio is 20% Protein ratio is 20% Fat ratio is 60%.

**Table. 2 Primers List.**

| **S.No** | **Name** | **Forward Primer (5' TO 3')** | **Reverse Primer (5' to 3')** |
| --- | --- | --- | --- |
| 1 | **β-actin** | CCTTCCTGGGTATGGAATCCTG | GCTAGGAGCCAGGGCAGTAA |
| 2 | **SIRT1** | ACACACAAAATCCAGCAACTC | GATGCTGTTGCAAAGGAACCA |
| 3 | **DNMT1** | GGTTCTGCGCGGGGACAGAC | CCGGCAACATGGCCTCAGGG |
| 4 | **DNMT3a** | ACGCCAAAGAAGTGTCTGCT | CTTGGCTATTCTGCCGTGTT |
| 5 | **DNMT3b** | TCTGGCCACCTTCAATAAGC | TGGTCCTCCAGTGAGTCTCC |
| 6 | **HDAC1** | CCGGCTTCTGTTACGTCAAT | CATGACCCGGTCTGTGGTAT |
| 7 | **AMPK-β1** | TACGTCACCACCCTCCTCTA | CCATCCGATTTCTGGCTGTG |
| 8 | **AMPK-β2** | TGATGTGTGTGTCAGAGCCT | CTTCCGGTACACCCATTCCT |
| 9 | **Adiponectin** | GAGACGCAGGTGTTCTTGGT | TTCTCCGGGCTCTCCTTTCC |
| 10 | **PGC-1α** | TGGAGTGACATAGAGTGTGCTG | CTGATCCTGTGGGTGTGGTT |
| 11 | **PPAR-α** | AACCGGAACAAATGCCAGTA | CAGGTAGGCTTCGTGGATTCTC |
| 12 | **SREBP-1c** | CGCTACCGTTCCTCTATCAATGAC | AGTTTCTGGTTGCTGTGCTGTAAG |
| 13 | **FAS** | CCACCGCCTACTACTCCTTA | CTGCTCAAACGATGTGTCTC |
| 14 | **SCD 1** | AAGGTGCCCCTCTATCTGGAA | AAATATCCCCCAGAGCAAGGTG |
| 15 | **ACOX2** | CTTGATCCGGAAGGATGCCA | TGCTTCTCGGTCCCAAATCC |
| 16 | **CPT1** | GCAGCTCGCACATTACAAGG | CTCTGCTCTGCCGTTGACTT |
| 17 | **Pyruvatekinase** | CCAAGGGACTGAGATACGA | AGGTCCACCTCAGTGTTTGG |
| 18 | **Glucokinase** | CAGTGGAGCGTGAAGACAAA | CTTGGTCCAATTGAGGAGGA |
| 19 | **G6Pase** | AGCTCCGTGCCTCTGATAAA | AAAGTGAGCCGCAAGGTAGA |
| 20 | **PEPCK** | CCCAGGAGTCACCATCACTT | TTCGTAGACAAGGGGGACAC |
| 21 | **Leptin** | CCAGGATGACACCAAAACCC | ACTCAGAATGGGGTGAAGCC |
| 22 | **PPAR-γ** | CACAATGCCATCAGGTTTGG | GCGGGAAGGACTTTATGTATGAG |

**Table. 3 Successful Mating of Male Parents.**

| **Animal Group** | **Number of Males Mated** | **Copulation Plugs Formed** | **No of Males Produced Pups Successfully** |
| --- | --- | --- | --- |
| **Control** | 6 | 6 | 6 |
| **HF** | 6 | 5 | 5 |
| **HFCR-I** | 6 | 4 | 4 |
| **HFCR-II** | 5 | 5 | 5 |

**Table. 4 Litter size and Sex ratio of the offspring.**

| **`** | **Number of Pups Born** | **Number of Male Pups** | **Number of Female Pups** |
| --- | --- | --- | --- |
| **Control** | 10±0.37 | 5±0.37 | 5±0.37 |
| **HF** | 11.33±0.56 | 5.50±0.43 | 5.833±0.31 |
| **HFCR-I** | 9.83±0.48 | 6±0.37 | 3.833±0.31** |
| **HFCR-II** | 11±0.58 | 6.33±0.42 | 5±0.37 |

Data was presented as mean±SEM where *P < 0.1; **P < 0.01; ***P < 0.001 statistically significance compared to their respective controls. The groups were analysed using one-way ANOVA with Dunnett’s post hoc test.

**Table. 5 Organ and WAT weights of the offspring.**

**a**

| **Organ weights (g)** | **Male Offspring** | | | |
| --- | --- | --- | --- | --- |
|  | **C** | **HF** | **HFCR-I** | **HFCR-II** |
| **Liver** | 11.90±0.37 | 14.62±0.54* | 14.59±0.84* | 12.88±0.17 |
| **Lungs** | 1.632±0.13 | 2.100±0.00 | 2.134±0.07 | 1.868±0.09 |
| **Heart** | 1.343±0.08 | 1.542±0.01 | 1.480±0.14 | 1.438±0.10 |
| **Brain** | 1.196±0.09 | 1.147±0.09 | 1.153±0.02 | 1.163±0.10 |
| **Kidneys** | 2.608±0.08 | 2.841±0.02 | 2.912±0.03 | 2.760±0.12 |
| **Pancreas** | 0.6623±0.09 | 0.9110±0.09 | 0.9505±0.15 | 0.8248±0.04 |
| **Spleen** | 0.5603±0.04 | 0.6723±0.00 | 0.6530±0.01 | 0.6038±0.02 |
| **Adiposity Index (%)** | 5.657±0.05 | 7.281±0.30** | 10.45±0.30*** | 6.607±0.33 |

**b**

| **Organ weights (g)** | **Female Offspring** | | | | | | |
| --- | --- | --- | --- | --- | --- | --- | --- |
|  | **C** | | **HF** | | **HFCR-I** | | **HFCR-II** |
| **Liver** | 5.83±0.27 | | 7.35±0.32** | | 7.25±0.23** | | 6.50±0.12 |
| **Lungs** | 1.186±0.01 | 1.271±0.08 | | 1.237±0.04 | | 1.151±0.03 | |
| **Heart** | 0.8603±0.03 | | 0.9598±0.02 | | 0.9954±0.04 | | 0.9170±0.02 |
| **Brain** | 1.531±0.06 | | 1.404±0.07 | | 1.455±0.03 | | 1.506±0.02 |
| **Kidneys** | 1.574±0.03 | | 1.613±0.02 | | 1.794±0.06** | | 1.560±0.03 |
| **pancreas** | 0.6725±0.07 | | 0.7228±0.05 | | 0.7093±0.10 | | 0.6780±0.05 |
| **Spleen** | 0.4145±0.00 | | 0.4458±0.01 | | 0.4573±0.02 | | 0.4245±0.02 |
| **Adiposity Index (%)** | 7.567±0.59 | | 9.520±0.16 | | 11.36±0.81** | | 8.806±0.72 |

Data was presented as mean±SEM where *P < 0.1; **P < 0.01; ***P < 0.001 statistically significance compared to their respective controls. The groups were analysed using one-way ANOVA with Dunnett’s post hoc test.

**Table. 6 Comparative analysis between different diet groups of male and female offspring**

| **Parameters** | **Male offspring** | | | | **Female offspring** | | | |
| --- | --- | --- | --- | --- | --- | --- | --- | --- |
|  | **C** | **HF** | **HFCR-I** | **HFCR-II** | **C** | **HF** | **HFCR-I** | **HFCR-II** |
| **Hepatocyte fat (%)** | 2.6±1.2 | 48.0±8.6 | 54.0±6.0 | 20.6±5.3 | 5.3±2.2 | 55.1±8.1 | 60.0±9.1 | 32.6±3.6* |
| **TC (mg/dL)** | 87.48±0.41 | 98.60±0.58 | 102.7±0.52 | 86.94±0.80 | 82.86±2.23 | 95.42±0.39 | 100.3±0.50 | 82.16±1.2 |
| **TG (mg/dL)** | 79.63±2.41 | 99.00±1.78 | 102.3±1.53 | 82.61±2.00 | 63.97±2.19 | 95.21±1.77 | 96.27±1.82 | 71.15±1.79 |
| **HDL (mg/dL)** | 75.72±2.14 | 62.67±1.05 | 95.34±1.51 | 79.18±1.10 | 77.21±7.2 | 56.07±2.19 | 79.59±1.80* | 70..93±2.52 |
| **FAS expression** | 1±0 | 1.76±0.04 | 1.86±0.04 | 1.17±0.08 | 1±0 | 2.76±0.13*** | 2.64±0.13*** | 1.19±0.02 |
| **SCD1 expression** | 1±0 | 2.38±0.03 | 2.82±0.02 | 1.19±0.083 | 1±0 | 4.32±0.11*** | 5.12±0.23*** | 2.62±0.14* |
| **Acox2 expression** | 1±0 | 0.82±0.03 | 0.67±0.03 | 0.90±0.02 | 1±0 | 0.87±0.03 | 0.50±0.04* | 1.07±0.02** |
| **CPT1 expression** | 1±0 | 0.79±0.04 | 0.41±0.03 | 0.92±0.02 | 1±0 | 0.77±0.06 | 0.46±0.02 | 0.97±0.05 |
| **Insulin (mg/dL)** | 13.15±0.39 | 15.72±0.52 | 17.48±0.41 | 13.64±0.46 | 13.40±0.34 | 20.30±0.53*** | 22.36±0.38*** | 14.66±0.43 |
| **GK expression** | 1±0 | 0.58±0.05 | 0.43±0.03 | 0.98±0.05 | 1±0 | 0.71±0.03 | 0.70±0.03*** | 0.91±0.02 |
| **PK expression** | 1±0 | 0.68±0.03 | 0.49±0.05 | 0.89±0.03 | 1±0 | 0.56±0.01 | 0.70±0.03*** | 0.92±0.02 |
| **G6-P expression** | 1±0 | 1.57±0.03 | 1.69±0.07 | 1.17±0.1 | 1±0 | 1.79±0.05 | 1.90±0.03 | 1.30±0.2 |
| **PEPCK expression** | 1±0 | 1.59±0.08 | 1.69±0.07 | 1.16±0.1 | 1±0 | 3.17±0.12*** | 3.06±0.21*** | 1.57±0.18 |

Data was presented as mean±SEM where *P < 0.1; **P < 0.01; ***P < 0.001 statistically significant between male vs females. The gene expression was normalized with β-actin and the groups were analysed using Two-way ANOVA.
